# Supplementary material for: Target Enrichment Enables the Discovery of lncRNAs with Somatic Mutations or Altered Expression in Paraffin-Embedded Colorectal Cancer Samples
Source: Cancers (Basel). 2020 Oct 1;12(10):2844. doi: 10.3390/cancers12102844 (PMC7650602; doi:10.3390/cancers12102844)
Supplement: Supplementary file 1 [file cancers-12-02844-s001.zip › cancers-925673 Supplementary/Supplementary.pdf]

Supplementary Materials: Target Enrichment Enables the Discovery of lncRNAs with Somatic Mutations or Altered Expression in Paraffin-Embedded Colorectal Cancer Samples

Susana Iraola-Guzmán, Anna Brunet-Vega, Cinta Pegueroles, Ester Saus, Hrant Hovhannisyan, Alex Casalots, Carles Pericay and Toni Gabaldón

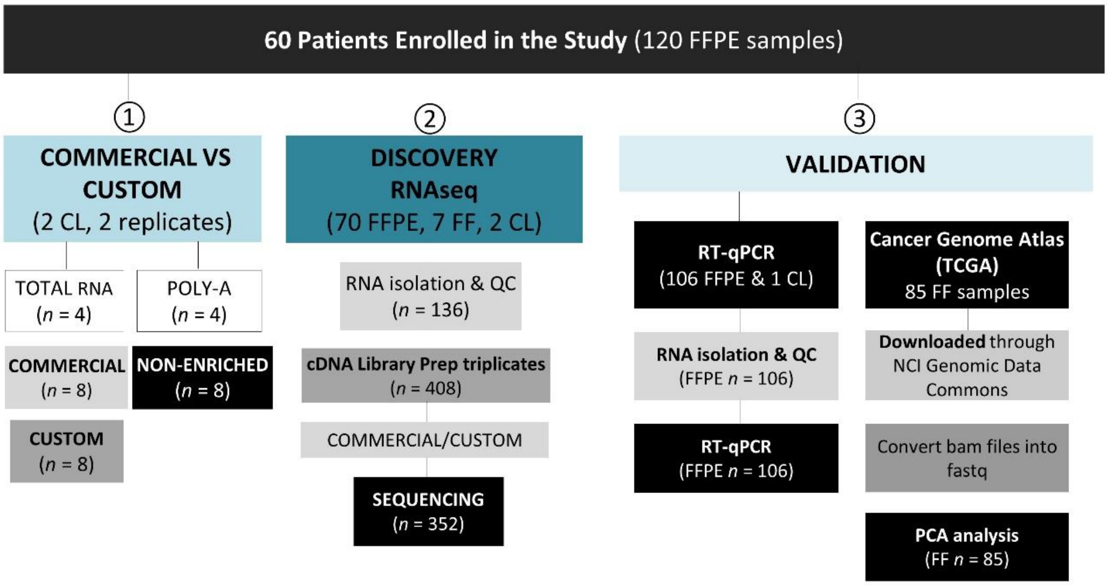

Figure S1. Schematic representation of the study showing the three main steps and the samples used. A total of 120 FFPE samples from 60 different patients were analyzed in the study: 70 FFPE samples in the discovery phase and 106 in the validation phase. Seven fresh-frozen samples were also included in the discovery phase, and two CRC cell lines were used in the three study phases. Finally, information obtained from 85 FF samples from the Cancer Genome Atlas (TCGA) was also used in the validation step.

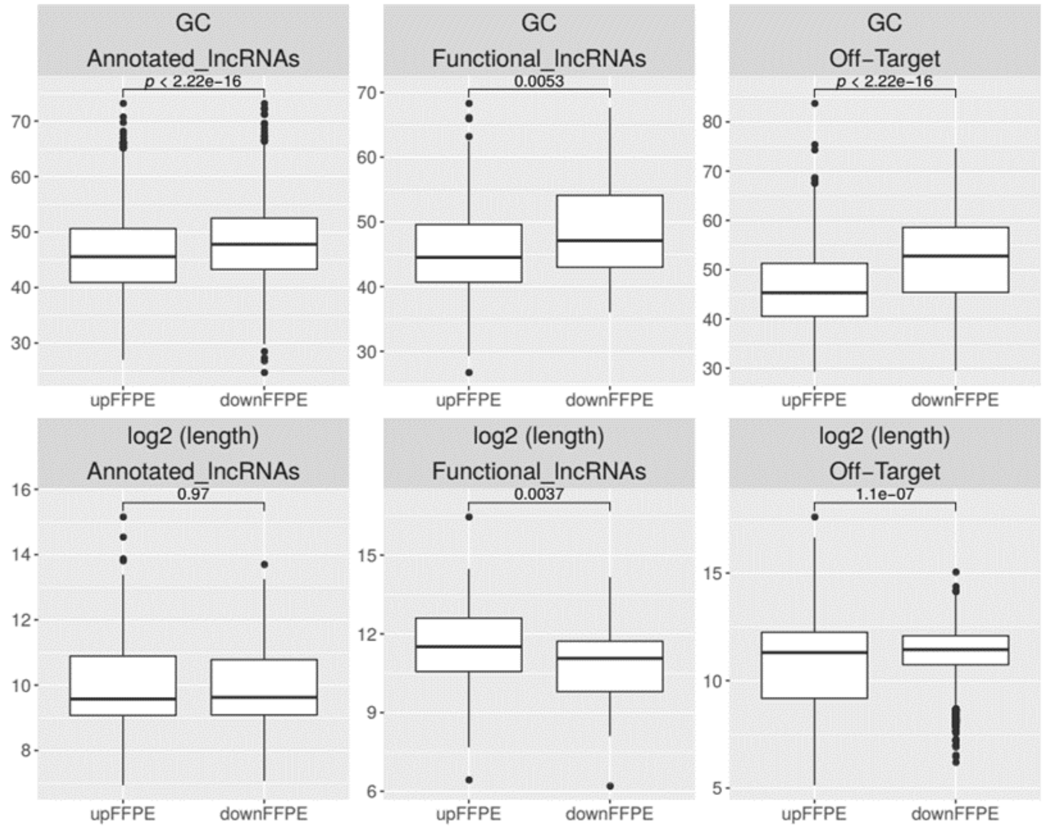

Figure S2. Characteristics of lncRNAs that are detected as DE in either the FFPE (Formalin-fixed paraffin-embedded)or TCGA datasets. The boxplots show GC content (or guanine-cytosine content) and log2 length levels for DE lncRNAs. We calculated log2 FoldChange values between FFPE and FF samples after filtering out genes in which more than 75% of the samples had normalized counts lower than 1. LncRNAs were classified as down and up-regulated on the basis of the log2 FoldChange values, using zero as a threshold. UpFFPE label refers to genes up-regulated in FFPE and down-regulated in FF samples and downFFPE label refers to genes down-regulated in FFPE and up-regulated in FF samples.

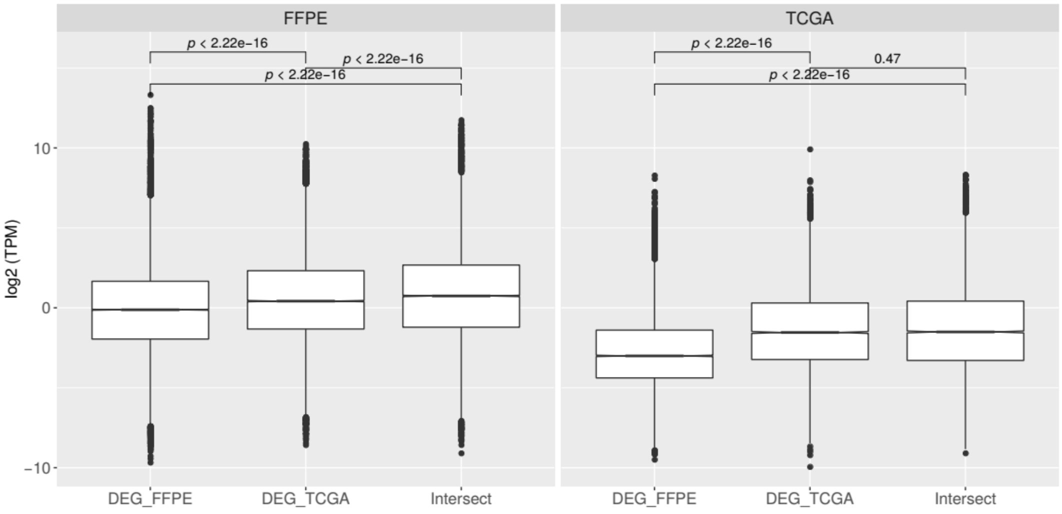

Figure S3. Comparison of gene expression levels for lncRNAs detected as differentially expressed (DE) in FFPE, TCGA, and both datasets (intersect). For each gene, we retrieved gene expression levels from FFPE (left) and TGCA (right) samples independently.

A

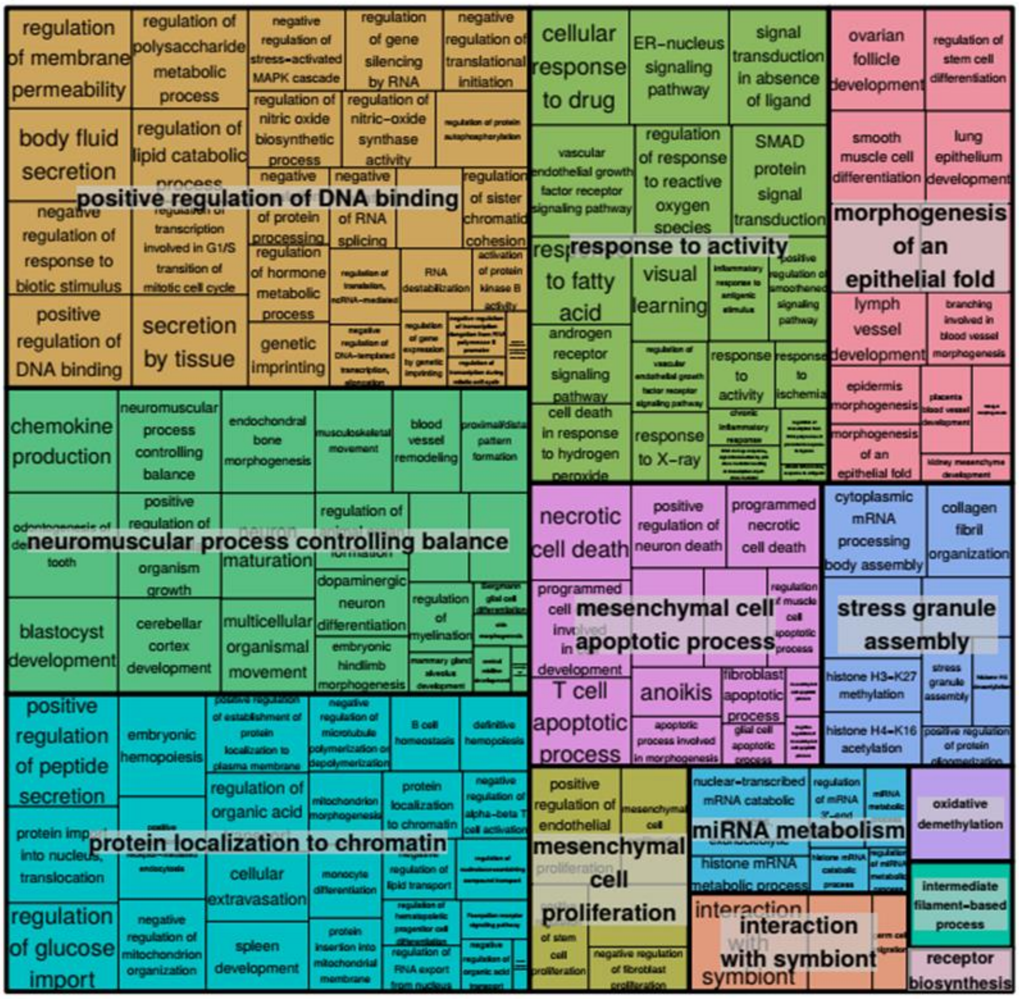
positive regulation of DNA binding
regulation of membrane permeability
regulation of polysaccharide metabolic process
negative regulation of stress-activated MAPK cascade
regulation of gene silencing by RNA
negative regulation of translational initiation
body fluid secretion
regulation of lipid catabolic process
regulation of nitric oxide biosynthetic process
regulation of nitric-oxide synthase activity
regulation of sister chromatid cohesion
negative regulation of response to biotic stimulus
regulation of transcription involved in G1/S transition of mitotic cell cycle
regulation of hormone metabolic process
RNA destabilization
activation of protein kinase B activity
positive regulation of DNA binding
secretion by tissue
genetic imprinting
cellular response to drug
ER-nucleus signaling pathway
signal transduction in absence of ligand
vascular endothelial growth factor receptor signaling pathway
regulation of response to reactive oxygen species
SMAD protein signal transduction
response to activity
response to fatty acid
visual learning
androgen receptor signaling pathway
response to activity
response to ischemia
cell death in response to hydrogen peroxide
response to X-ray
ovarian follicle development
regulation of stem cell differentiation
smooth muscle cell differentiation
lung epithelium development
morphogenesis of an epithelial fold
lymph vessel development
branching involved in blood vessel morphogenesis
epidermis morphogenesis
morphogenesis of an epithelial fold
neuromuscular process controlling balance
chemokine production
neuromuscular process controlling balance
endochondral bone morphogenesis
musculoskeletal movement
blood vessel remodeling
proximal/distal pattern formation
positive regulation of organism growth
maturation
dopaminergic neuron differentiation
blastocyst development
cerebellar cortex development
multicellular organismal movement
embryonic hindlimb morphogenesis
regulation of myelination
necrotic cell death
positive regulation of neuron death
programmed necrotic cell death
mesenchymal cell apoptotic process
T cell apoptotic process
anoikis
fibroblast apoptotic process
cytoplasmic mRNA processing body assembly
collagen fibril organization
stress granule assembly
histone H3-K27 methylation
histone H4-K16 acetylation
positive regulation of peptide secretion
embryonic hemopoiesis
B cell homeostasis
definitive hemopoiesis
protein localization to chromatin
regulation of organic acid transport
mitochondrion morphogenesis
protein localization to chromatin
negative regulation of alpha-beta T cell activation
protein import into nucleus, translocation
cellular extravasation
monocyte differentiation
regulation of lipid transport
regulation of glucose import
negative regulation of mitochondrion organization
spleen development
protein insertion into mitochondrial membrane
regulation of RNA export from nucleus
positive regulation of endothelial cell proliferation
mesenchymal cell proliferation
negative regulation of fibroblast proliferation
nuclear-transcribed mRNA catabolic process
miRNA metabolism
histone mRNA metabolic process
oxidative demethylation
interaction with symbiont
intermediate filament-based process
receptor biosynthesis

B

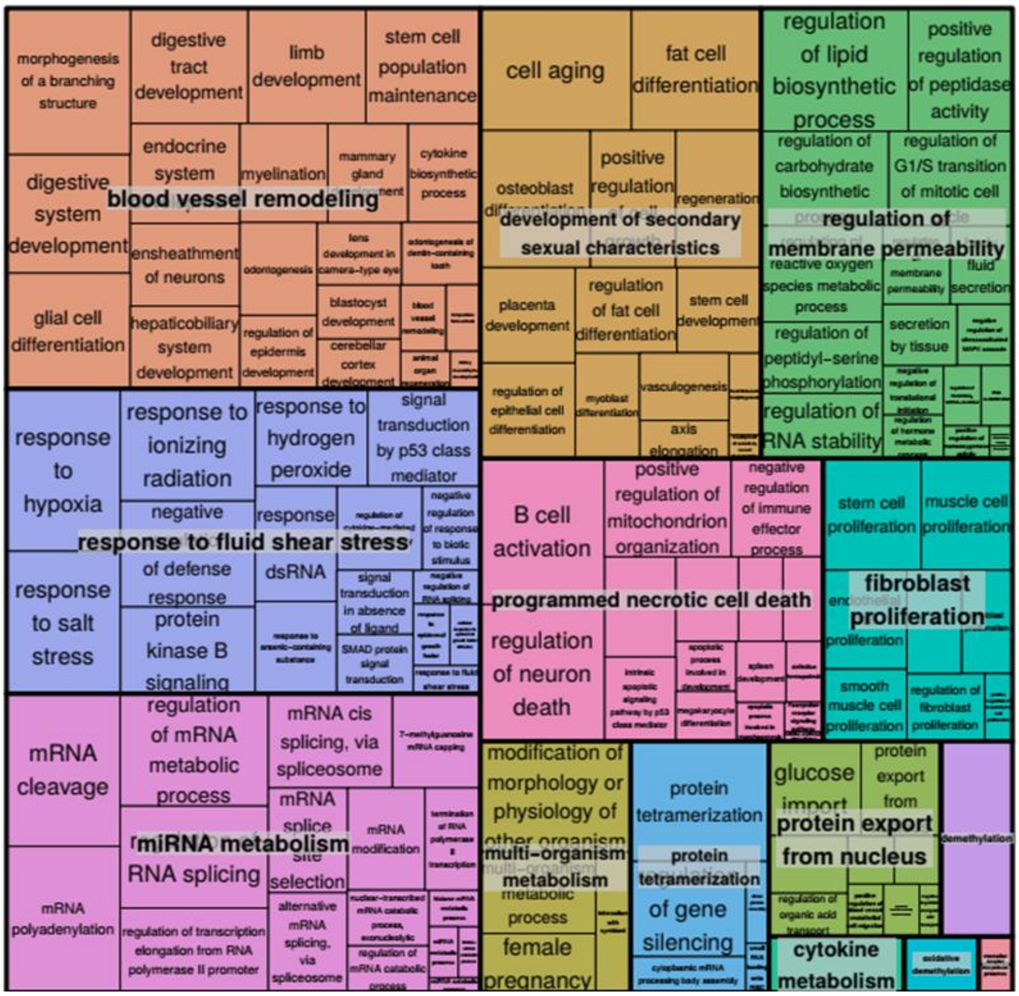
blood vessel remodeling
morphogenesis of a branching structure
digestive tract development
limb development
stem cell population maintenance
digestive system development
endocrine system
myelination
mammary gland
cytokine biosynthetic process
ensheathment of neurons
odontogenesis
glial cell differentiation
hepaticobiliary system development
regulation of epidermis development
blastocyst development
cerebellar cortex
response to fluid shear stress
response to hypoxia
response to ionizing radiation
response to hydrogen peroxide
signal transduction by p53 class mediator
of defense response
response to salt stress
protein kinase B signaling
dsRNA
SMAD protein signal transduction
miRNA metabolism
mRNA cleavage
regulation of mRNA metabolic process
mRNA cis splicing, via spliceosome
RNA splicing
mRNA modification
mRNA polyadenylation
regulation of transcription elongation from RNA polymerase II promoter
alternative mRNA splicing, via spliceosome
development of secondary sexual characteristics
cell aging
fat cell differentiation
osteoblast
positive regulation
regeneration
placenta development
regulation of fat cell differentiation
stem cell development
regulation of epithelial cell differentiation
vasculogenesis
programmed necrotic cell death
B cell activation
positive regulation of mitochondrion organization
negative regulation of immune effector process
regulation of neuron death
multi-organism metabolism
modification of morphology or physiology of other organism
protein tetramerization
of gene silencing
female pregnancy
regulation of membrane permeability
regulation of lipid biosynthetic process
positive regulation of peptidase activity
regulation of carbohydrate biosynthetic
regulation of G1/S transition of mitotic cell
reactive oxygen species metabolic process
fluid secretion
secretion by tissue
regulation of peptidyl-serine phosphorylation
regulation of RNA stability
fibroblast proliferation
stem cell proliferation
muscle cell proliferation
smooth muscle cell proliferation
regulation of fibroblast proliferation
protein export from nucleus
glucose import
demethylation
cytokine metabolism
oxidative demethylation

C

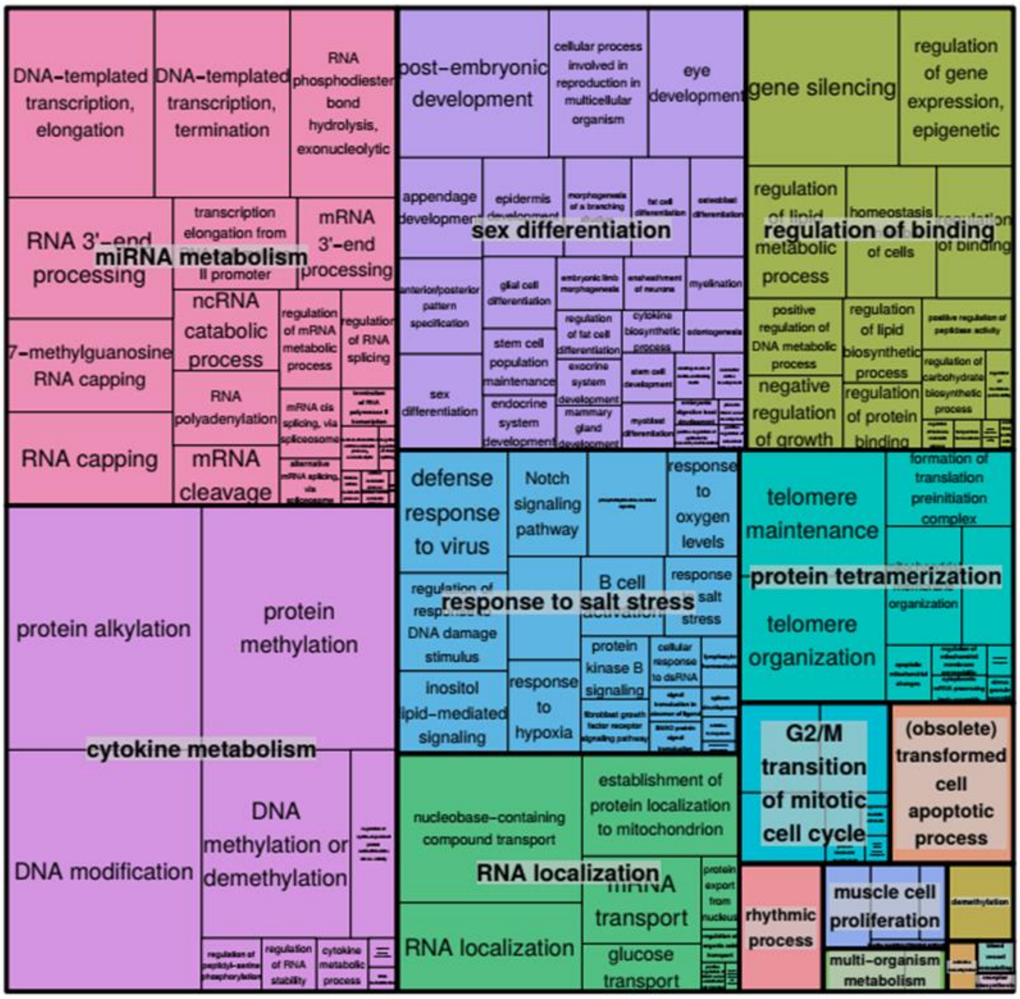

Figure S4. Functional enrichment analysis for (**A**) FFPE DE list, (**B**) TCGA DE list and (**C**) overlapped DE list. REVIGO Gene Ontology treemap_overlapped. See Table S6 for details within each box.

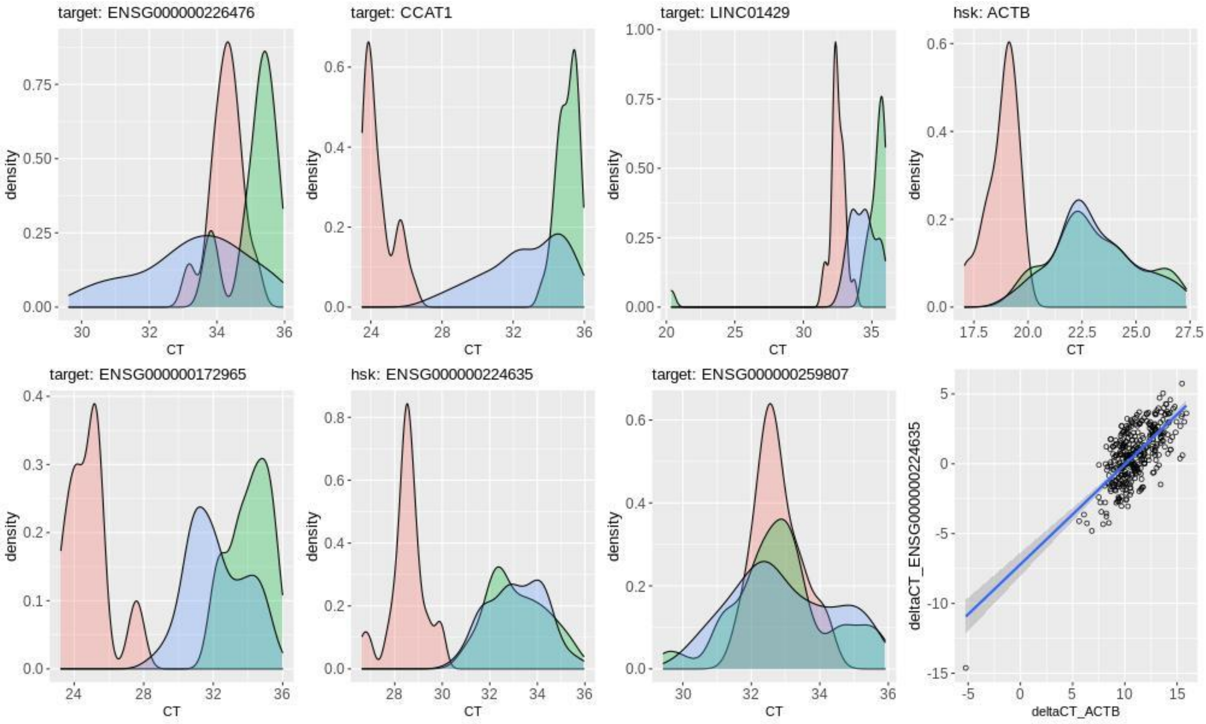

Figure S5. CT (cycle threshold) levels of the five DE lncRNAs selected for RT-qPCR validation (targets) and its respective housekeeping (hsk) beta actin (ACTB) gene for CRC cell line HCT116 (red), FFPE normal (green) and FFPE tumoral (blue) samples and dot plot showing the relative expression levels (deltaCT) of the two housekeeping genes included in the validation analysis by RT-qPCR. The relative expression of each target lncRNA was calculated using the 'Delta Ct method' (Ct target-Ct housekeeping), where the two housekeeping genes were *ACTB* and *ENSG00000224635*.

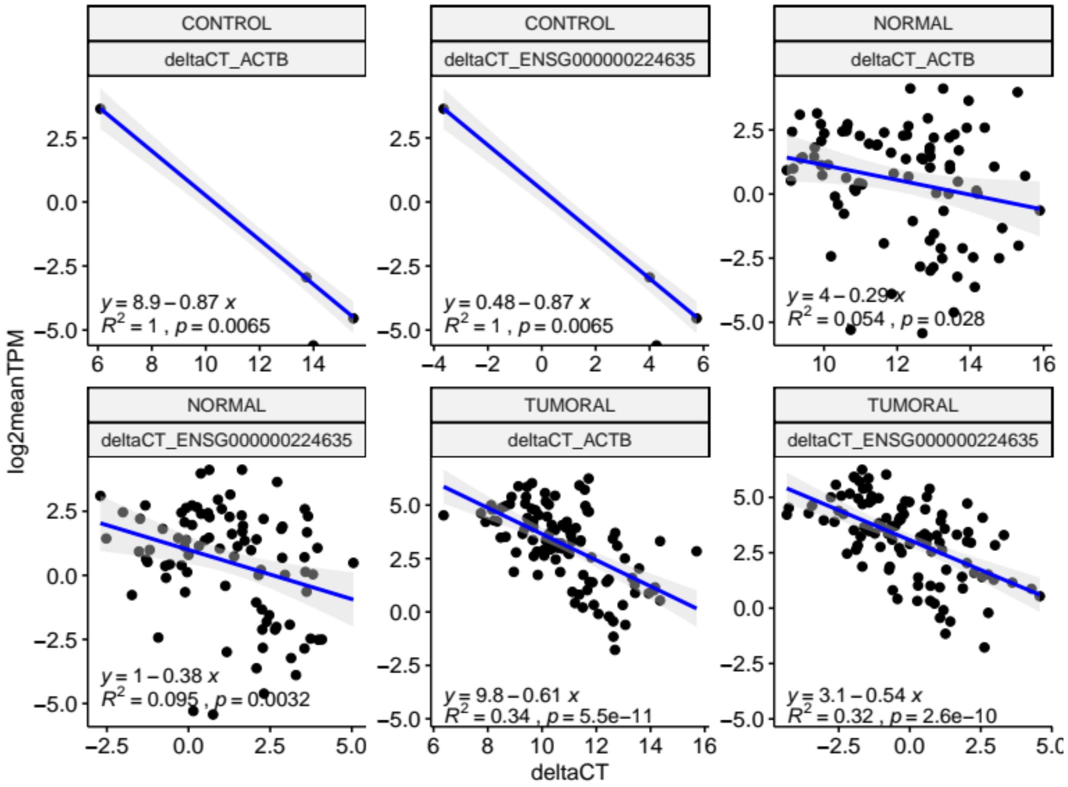

Figure S6. Gene expression levels between RNA-Seq (log2(TPM)) and RT-qPCR (Delta Ct values) for normal, tumoral and control (CRC cell line HCT116) samples. DeltaCT was computed for the two housekeeping genes independently. Each dot shows gene expression values of each target lncRNA in

each sample (FFPE or HCT116 cell line). Pearson correlations were estimated using stat_cor function from R, being R^2 the squared correlation coefficient, and x and y are respectively the independent and dependent variables from the regression line equation.

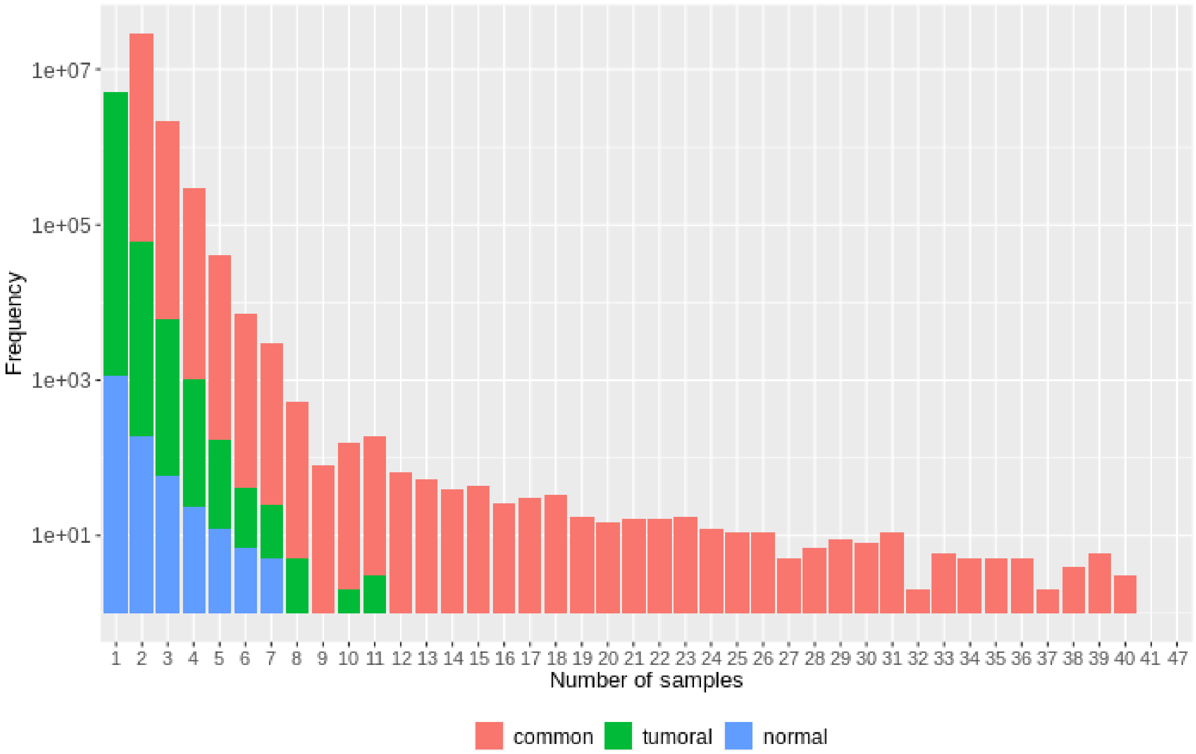

Figure S7. Frequency distribution of the number of somatic variants found. X-axis designates the number of samples in which a given variant is found.

Table S1 (separate Excel file "Supplementary Tables S1-S10"): Differentially expressed (DE) lncRNAs in enriched FFPE samples using CoLong design. It includes Ensembl gene ID, description, HGNC gene name, several parameters reported by Deseq2 software (baseMean, log2FoldChange, lfcSE, stat, p-value, padj), gene biotype, functionality (functional or annotated), SpliceMap (presence of evolutionary conserved splice sites), CPAT (yes: predicted as coding according to CPAT software), CPC (yes: predicted as coding according to CPC software), ultraconserved (indicates whether lncRNA contain any of the ultraconserved elements from https://users.soe.ucsc.edu/~jill/ultra.html [3]), OverlapProtCod_Ensr96 (overlap with protein coding genes in Ensembl r96), CLC (LncRNAs included in the Cancer LncRNA Census list [2]) and DE.in.TCGA (yes: lncRNAs also DE in TCGA samples, which are highlighted in grey).

Table S2 (separate Excel file "Supplementary Tables S1-S11"): Differentially expressed (DE) lncRNAs in TCGA samples. It includes Ensembl gene ID, several parameters reported by Deseq2 software (baseMean, log2FoldChange, lfcSE, stat, *p*-value, padj), gene biotype, functionality (functional or annotated, see above), SpliceMap (presence of evolutionary conserved splice sites), CPAT (yes: predicted as coding according to CPAT software), CPC (yes: predicted as coding according to CPC software), ultraconserved (indicates whether lncRNA contain any of the ultraconserved elements from https://users.soe.ucsc.edu/~jill/ultra.html [3]), OverlapProtCod (overlap with protein coding genes in Ensembl r96), CLC (LncRNAs included in the Cancer LncRNA Census list [2]) and DE.in.FFPE (yes: lncRNAs also DE in FFPE samples, which are highlighted in grey).

Table S3 (separate Excel file "Supplementary Tables S1-S11"): LnCompare summarized results after comparing two lists of lncRNAs. Qualitative and quantitative features significantly enriched (*p*-adjusted < 0.05) when comparing OverlappedvsEnr (lncRNAs DE in overlapped and TCGA vs lncRNAs DE in FFPE enriched samples only), OverlappedvsTCGA (lncRNAs DE in overlapped and

TCGA vs lncRNAs DE in TCGA samples only) and EnrvsTCGA (lncRNAs DE in enriched FFPE samples only vs lncRNAs DE in TCGA samples only). LnCompare was accessed 23 April 2020.

Table S4 (separate Excel file "Supplementary Tables S1-S11"): LnCompare summarized results when comparing a list of lncRNAs and the rest of lncRNAs annotated in ENCODE as background: qualitative and quantitative features significantly enriched (*p*-adjusted < 0.05). LnCompare was accessed 14 February 2020 for FFPE, 19 May 2020 for TCGA samples and 21 May 2020 for the overlapped list of DE lncRNAs.

Table S5 (separate Excel file "Supplementary Tables S1-S11"): Experimentally validated interactions of differentially expressed (DE) lncRNAs in NPInter v4.0 database [1] for FFPE, TCGA and overlapped DE lncRNAs.

Table S6 (separate Excel file "Supplementary Tables S1-S11"): Results from the GO term enrichment analysis as reported by Revigo software; plots are shown in Figure S4.

Table S7 (separate Excel file "Supplementary Tables S1-S11"): Complete list of somatic variants found in tumoral samples. It contains the chromosome, position (GRCh38 coordinates), reference allele, alternative allele, Ensembl gene ID, gene name, total_Individuals (number of individuals with the variant), total_relapsed (number of relapsed individuals with the variant), total_nonRelapsed (number of non-relapsed individuals with the variant), relative abundance in relapsed samples and relative abundance in non-relapsed samples label (functional or annotated, see above), DE_FFPE (differentially expressed in FFPE samples), DE_TGCA (differentially expressed in TCGA samples), DE_overlap (differentially expressed in both FFPE and TCGA samples) The eleven most abundant variants are highlighted in gray.

Table S8 (separate Excel file "Supplementary Tables S1-S11"): Demographic and clinicopathological layout, summary of the type of samples analyzed and techniques applied. According to selection criteria we included FFPE samples from a total of 60 patients diagnosed from five to ten years ago, at stage II CRC. Finally, we balanced the cohort considering demographic information (age, gender). We analyzed 37 patients and 53 patients out of the 60 by RNAseq and RT-qPCR, respectively. Fresh-frozen samples were selected based on availability. CRC stages (I–IV); MSI (microsatellite, instable); MSS (microsatellite, stable).

Table S9 (separate Excel file "Supplementary Tables S1-S11"): List of lncRNAs included in the CoLong design. If present in Ensembl releases 86 and 99, their biotype is indicated. LncRNAs are classified as functional (i.e., those with experimentally validated functions, mostly CRC related) and annotated (i.e., those annotated in Ensembl but with no further knowledge of their functionality).

Table S10 (separate Excel file "Supplementary Tables S1-S11"): Detailed information of the functionally characterized lncRNA. Columns indicate, in this order: gene ID, HGNC symbol, description, DOI and database with functional information. Those lncRNAS specifically related to colorectal cancer are labelled as CRC.

Table S11 (separate Excel file "Supplementary Tables S1-S11"): Primers for RT-qPCR assays. Sequences of the primers (forward and reverse) and probes included in the RT-qPCR validation. Assays were designed in spanning-intron regions, of around 100 bp, common to alternative transcripts validated by RNAseq data, preferentially with no information reported about SNPs.

References

1. Teng, X.; Chen, X.; Xue, H.; Tang, Y.; Zhang, P.; Kang, Q.; Hao, Y.; Chen, R.; Zhao, Y.; He, S. NPInter v4.0: An integrated database of ncRNA interactions. *Nucleic Acids Res*. **2020**, *48*, D160–D165, doi:10.1093/nar/gkz969.

2. Carlevaro-Fita, J.; Lanzós, A.; Feuerbach, L.; et al. Cancer LncRNA Census reveals evidence for deep functional conservation of long noncoding RNAs in tumorigenesis. *Commun Biol* **2020**, *3*, 56, doi:10.1038/s42003-019-0741-7
3. Bejerano, G.; Pheasant, M.; Makunin, I.; Stephen, S.; Kent, W.J.; Mattick J.S.; Haussler, D. Ultraconserved Elements in the Human Genome. *Science* **2004**, *304*, 1321–1325, doi: 10.1126/science.1098119

© 2020 by the authors. Licensee MDPI, Basel, Switzerland. This article is an open access article distributed under the terms and conditions of the Creative Commons Attribution (CC BY) license (http://creativecommons.org/licenses/by/4.0/).
